# The Treatment of Early Cancer of the Larynx

**Published:** 1902-12-06

**Authors:** 


### THE TREATMENT OF EARLY CANCER OF THE LARYNX.

By the relation of two successful cases Dr. Eugene S. Yonge[1] emphasises the advantages of operation by thyrotomy in cases of early cancer of the larynx. He points out that in the majority of instances epithelioma is at first intrinsic, taking its origin from a vocal cord or ventricular band, and being confined, often for a considerable time, within the laryngeal cavity. The growth tends but little at first to invade and penetrate the cartilage, and spreads by preference upwards towards the superior aperture, or downwards towards the trachea. Commonly also, although not universally, involvement of the lymphatic glands is very late in making its appearance, not occurring as a rule so long as the growth remains intrinsic. Whatever may be the reason for this peculiar isolation of the larynx, its result is that intra-mural neoplasms are thereby rendered more amenable to operative treatment than would otherwise be the case. Hence the very great importance of early diagnosis, for it is at this time, when the mischief is still purely local, that the comparatively simple procedure of thyrotomy with excision of the diseased tissue, including the cartilage if necessary, may be employed, often with brilliant results. By this procedure not only may the growth be radically extirpated but a not inconsiderable restoration of the voice may be obtained. The cases given are certainly very striking. They were operated on more than a year ago and so far they are still free from signs of recurrence. In regard to diagnosis Dr. Yonge says that the most characteristic early symptom is a persistent dry hoarseness, especially if occurring in a person over 50 years of age. Such symptoms as pain on swallowing, glandular enlargement, blood-spitting, and cachexia, are really indicative of the later stages of epithelioma of the larynx, and are generally to be regarded rather as contra-indications to the operation. In some cases the removal of a piece of the growth for microscopical examination may be advisable, but if this is done it is desirable that the piece removed should not be too minute. The fragment should include some of the deeper portions of the growth. An important early symptom is a sluggishness in the movement of one or other cord not amounting to paralysis, while the involvement of the arytenoid region by the growth should give rise to suspicion as to its malignant nature. The operation is a simple one in comparison with removal of the larynx.

[1] Lancet, Nov. 15.

